# *catena*-Poly[[(2,9-dimethyl-1,10-phenanthroline-κ^2^ *N*,*N*′)cobalt(II)]-μ-malonato-κ^4^ *O* ^1^,*O* ^1′^:*O* ^3^,*O* ^3′^]

**DOI:** 10.1107/S1600536810038043

**Published:** 2010-09-30

**Authors:** Ling-Feng Qiu, Bai-Lu Zhou, Wei Xu

**Affiliations:** a Center of Applied Solid State Chemistry Research, Ningbo University, Ningbo 315211, People’s Republic of China

## Abstract

In the title compound, [Co(C_3_H_2_O_4_)(C_14_H_12_N_2_)]_*n*_, the Co^II^ ion is in a distorted octa­hedral coordination being chelated by a 2,9-dimethyl-1,10-phenanthroline mol­ecule (dmphen) and two carboxyl­ate groups of two malonate ligands The malonate ligand acts in a bridging mode, forming coordination chains along [100]. π–π stacking inter­actions between dmphen ligands [inter­planar distances = 3.414 (4) and 3.447 (4) Å] organize the coordination polymers into supra­molecular double chains.

## Related literature

For coordination polymers with dicarboxyl­ate ligands, see: Rao *et al.* (2004 ▶); Zheng *et al.* (2004 ▶).
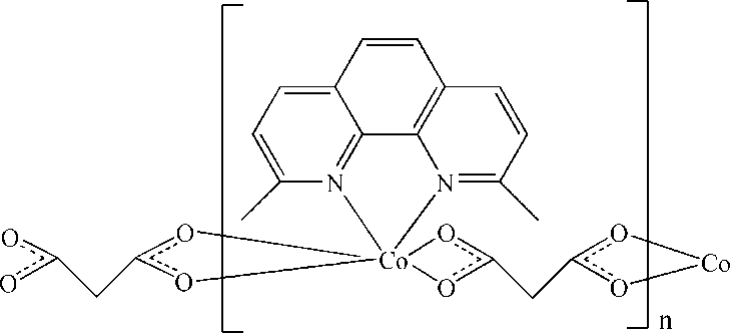


## Experimental

### 

#### Crystal data

[Co(C_3_H_2_O_4_)(C_14_H_12_N_2_)]
*M* *_r_* = 369.23
Triclinic, 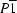

*a* = 6.8767 (14) Å
*b* = 9.5293 (19) Å
*c* = 11.149 (2) Å
α = 86.83 (3)°
β = 89.53 (3)°
γ = 89.52 (3)°
*V* = 729.4 (2) Å^3^
*Z* = 2
Mo *K*α radiation
μ = 1.20 mm^−1^
*T* = 295 K
0.33 × 0.11 × 0.07 mm

#### Data collection

Rigaku R-AXIS RAPID diffractometer
Absorption correction: multi-scan (*ABSCOR*; Higashi, 1995 ▶) *T*_min_ = 0.653, *T*_max_ = 0.782
7245 measured reflections
3309 independent reflections
2590 reflections with *I* > 2σ(*I*)
*R*_int_ = 0.032

#### Refinement

*R*[*F* ^2^ > 2σ(*F* ^2^)] = 0.048
*wR*(*F* ^2^) = 0.127
*S* = 1.06
3309 reflections
222 parameters
H-atom parameters constrained
Δρ_max_ = 0.58 e Å^−3^
Δρ_min_ = −0.40 e Å^−3^

### 

Data collection: *RAPID-AUTO* (Rigaku, 1998 ▶); cell refinement: *RAPID-AUTO*; data reduction: *RAPID-AUTO*; program(s) used to solve structure: *SHELXS97* (Sheldrick, 2008 ▶); program(s) used to refine structure: *SHELXL97* (Sheldrick, 2008 ▶); molecular graphics: *SHELXTL* (Sheldrick, 2008 ▶); software used to prepare material for publication: *SHELXL97*.

## Supplementary Material

Crystal structure: contains datablocks global, I. DOI: 10.1107/S1600536810038043/gk2299sup1.cif

Structure factors: contains datablocks I. DOI: 10.1107/S1600536810038043/gk2299Isup2.hkl

Additional supplementary materials:  crystallographic information; 3D view; checkCIF report

## Figures and Tables

**Table 1:** Selected bond lengths (Å)

Co1—O1	2.180 (3)
Co1—O2	2.145 (3)
Co1—O3^i^	2.229 (3)
Co1—O4^i^	2.126 (4)
Co1—N1	2.122 (3)
Co1—N2	2.103 (3)

Symmetry code: (i) 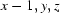.

**Fig. 1.**
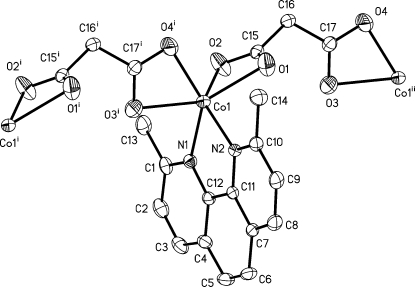
ORTEP view of the title compound. The displacement ellipsoids are drawn at the 30% probability level [symmetry code: (i) x - 1, y, z; (ii) x + 1, y, z].

**Fig. 2.**
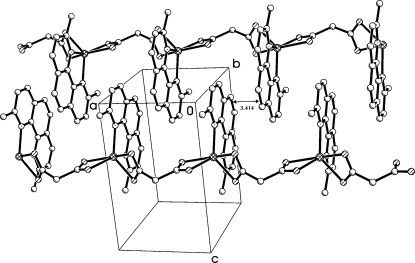
A double chain formed through π–π stacking interactions between dmphen ligands.

## supplementary crystallographic information

### Comment

Metal-phenanthroline complexes and their derivatives have attracted much attention because of their peculiar features. In turn dicarboxylate ligands play an important role in modern coordination chemistry and many complexes have been published with them as ligands (Rao *et al.*, 2004; Zheng *et al.*, 2004). The title complex, (I), was recently prepared and its crystal structure is reported here.

The crystal structure of the title compound consists of [Co(C_14_H_12_N_2_)(C_3_H_2_O_4_)]_n_ chains (Fig. 1). Each Co atom is surrounded by two nitrogen atoms of one 2,9-dimethyl-1,10-phenanthroline ligand and four oxygen atoms of two bis-chelating malonate anions to complete a seriously distorted octahedral coordination (Table 1). The malonate ligands bridge the Co atoms to form neutral one-dimensional chains [Co(C_14_H_12_N_2_)(C_3_H_2_O_4_)]_n_ along [100] with parallel orientated phen ligands at the same side. As shown in Fig. 2, through π-π stacking interactions the dmphen ligands of two adjacent coordination chains form supramolecular double chains. The interplanar distances between the neighbouring dmphen ligands are 3.414 (4) and 3.447 (4) Å.

### Experimental

Addition of 2.0 ml (1 *M*) NaOH to an aqueous solution of CoCl_2_.6H_2_O (0.238 g, 1.00 mmol) in 10.0 ml H_2_O produced a pink precipitate, which was centrifugated and washed with doubly destilled water for several times until no Cl^-^ anions were detectable. The fresh precipitate was then added to a stirred solution of malonic acid (0.104 g, 1.00 mmol) and 2,9-dimethyl-1,10-phenanthroline hydrate (0.226 g, 1 mmol) in CH_3_OH/H_2_O (1:1 30 ml). The red mixture was allowed to stand at room temperature and after several days, red plate-like crystals suitable for X-ray analysis were formed. grown by slow evaporation.

### Refinement

All H atoms were placed in geometrically calculated position (C-H = 0.93-0.97 Å) and refined in a riding model approximation with *U*_iso_(H) = 1.2 *U*_eq_(C).

### Crystal data

**Table:**

[Co(C_3_H_2_O_4_)(C_14_H_12_N_2_)]	*Z* = 2
*M**_r_* = 369.23	*F*(000) = 378
Triclinic, *P*1	*D*_x_ = 1.681 Mg m^−^^3^
Hall symbol: -P 1	Mo *K*α radiation, λ = 0.71073 Å
*a* = 6.8767 (14) Å	Cell parameters from 5667 reflections
*b* = 9.5293 (19) Å	θ = 3.5–27.5°
*c* = 11.149 (2) Å	µ = 1.20 mm^−^^1^
α = 86.83 (3)°	*T* = 295 K
β = 89.53 (3)°	Plate, red
γ = 89.52 (3)°	0.33 × 0.11 × 0.07 mm
*V* = 729.4 (2) Å^3^	

### Data collection

**Table:**

Rigaku R-AXIS RAPID diffractometer	3309 independent reflections
Radiation source: fine-focus sealed tube	2590 reflections with *I* > 2σ(*I*)
graphite	*R*_int_ = 0.032
Detector resolution: 0 pixels mm^-1^	θ_max_ = 27.5°, θ_min_ = 3.5°
ω scans	*h* = −8→8
Absorption correction: multi-scan (*ABSCOR*; Higashi, 1995)	*k* = −12→12
*T*_min_ = 0.653, *T*_max_ = 0.782	*l* = −14→14
7245 measured reflections	

### Refinement

**Table:**

Refinement on *F*^2^	Primary atom site location: structure-invariant direct methods
Least-squares matrix: full	Secondary atom site location: difference Fourier map
*R*[*F*^2^ > 2σ(*F*^2^)] = 0.048	Hydrogen site location: inferred from neighbouring sites
*wR*(*F*^2^) = 0.127	H-atom parameters constrained
*S* = 1.06	*w* = 1/[σ^2^(*F*_o_^2^) + (0.0568*P*)^2^ + 0.8449*P*] where *P* = (*F*_o_^2^ + 2*F*_c_^2^)/3
3309 reflections	(Δ/σ)_max_ = 0.015
222 parameters	Δρ_max_ = 0.58 e Å^−^^3^
0 restraints	Δρ_min_ = −0.40 e Å^−^^3^

### Special details

**Table:**

Geometry. All e.s.d.'s (except the e.s.d. in the dihedral angle between two l.s. planes) are estimated using the full covariance matrix. The cell e.s.d.'s are taken into account individually in the estimation of e.s.d.'s in distances, angles and torsion angles; correlations between e.s.d.'s in cell parameters are only used when they are defined by crystal symmetry. An approximate (isotropic) treatment of cell e.s.d.'s is used for estimating e.s.d.'s involving l.s. planes.
Refinement. Refinement of *F*^2^ against ALL reflections. The weighted *R*-factor *wR* and goodness of fit *S* are based on *F*^2^, conventional *R*-factors *R* are based on *F*, with *F* set to zero for negative *F*^2^. The threshold expression of *F*^2^ > σ(*F*^2^) is used only for calculating *R*-factors(gt) *etc*. and is not relevant to the choice of reflections for refinement. *R*-factors based on *F*^2^ are statistically about twice as large as those based on *F*, and *R*- factors based on ALL data will be even larger.

### Fractional atomic coordinates and isotropic or equivalent isotropic displacement parameters (Å^2^)

**Table:**

	*x*	*y*	*z*	*U*_iso_*/*U*_eq_	
Co1	0.23932 (7)	0.27775 (5)	0.73432 (4)	0.03063 (16)	
N1	0.2539 (4)	0.0677 (3)	0.6805 (2)	0.0283 (6)	
N2	0.2379 (4)	0.1661 (3)	0.9024 (2)	0.0264 (5)	
C1	0.2526 (5)	0.0223 (4)	0.5691 (3)	0.0346 (7)	
C2	0.2653 (5)	−0.1224 (4)	0.5494 (4)	0.0435 (9)	
H2A	0.2659	−0.1521	0.4713	0.052*	
C3	0.2765 (5)	−0.2181 (4)	0.6433 (4)	0.0439 (9)	
H3A	0.2860	−0.3133	0.6297	0.053*	
C4	0.2737 (5)	−0.1737 (3)	0.7614 (3)	0.0347 (7)	
C5	0.2824 (5)	−0.2671 (4)	0.8659 (4)	0.0437 (9)	
H5A	0.2923	−0.3633	0.8571	0.052*	
C6	0.2766 (5)	−0.2182 (4)	0.9773 (4)	0.0413 (9)	
H6A	0.2831	−0.2812	1.0439	0.050*	
C7	0.2604 (5)	−0.0702 (3)	0.9941 (3)	0.0322 (7)	
C8	0.2485 (5)	−0.0137 (4)	1.1072 (3)	0.0396 (8)	
H8A	0.2515	−0.0724	1.1766	0.048*	
C9	0.2326 (5)	0.1280 (4)	1.1146 (3)	0.0382 (8)	
H9A	0.2249	0.1660	1.1896	0.046*	
C10	0.2276 (5)	0.2173 (3)	1.0107 (3)	0.0312 (7)	
C11	0.2530 (4)	0.0242 (3)	0.8937 (3)	0.0266 (6)	
C12	0.2620 (4)	−0.0274 (3)	0.7747 (3)	0.0281 (7)	
C13	0.2325 (6)	0.1281 (5)	0.4669 (3)	0.0461 (9)	
H13A	0.1190	0.1850	0.4789	0.069*	
H13B	0.2202	0.0809	0.3935	0.069*	
H13C	0.3455	0.1867	0.4623	0.069*	
C14	0.2121 (6)	0.3725 (4)	1.0190 (3)	0.0424 (9)	
H14A	0.3391	0.4104	1.0304	0.064*	
H14B	0.1298	0.3933	1.0858	0.064*	
H14C	0.1574	0.4137	0.9462	0.064*	
O1	0.4924 (4)	0.3945 (4)	0.7882 (3)	0.0622 (9)	
O2	0.4560 (5)	0.3466 (4)	0.6052 (3)	0.0675 (9)	
O3	0.9214 (4)	0.2696 (3)	0.6983 (3)	0.0646 (9)	
O4	1.0687 (5)	0.4640 (4)	0.7095 (5)	0.0950 (15)	
C15	0.5528 (5)	0.4017 (3)	0.6837 (3)	0.0331 (7)	
C16	0.7380 (5)	0.4791 (4)	0.6502 (3)	0.0360 (8)	
H16A	0.7421	0.4975	0.5638	0.043*	
H16C	0.7359	0.5691	0.6869	0.043*	
C17	0.9200 (5)	0.3999 (4)	0.6886 (3)	0.0343 (7)	

### Atomic displacement parameters (Å^2^)

**Table:**

	*U*^11^	*U*^22^	*U*^33^	*U*^12^	*U*^13^	*U*^23^
Co1	0.0319 (2)	0.0274 (2)	0.0321 (3)	−0.00231 (17)	−0.00176 (17)	0.00297 (17)
N1	0.0266 (13)	0.0290 (13)	0.0292 (14)	−0.0007 (11)	0.0004 (11)	−0.0026 (11)
N2	0.0265 (13)	0.0237 (12)	0.0289 (14)	−0.0015 (10)	−0.0022 (10)	−0.0008 (10)
C1	0.0240 (15)	0.045 (2)	0.0350 (18)	−0.0042 (14)	0.0005 (13)	−0.0083 (15)
C2	0.040 (2)	0.052 (2)	0.040 (2)	−0.0062 (17)	0.0002 (16)	−0.0192 (18)
C3	0.0363 (19)	0.0362 (19)	0.061 (3)	−0.0042 (16)	−0.0004 (17)	−0.0184 (18)
C4	0.0284 (16)	0.0271 (16)	0.049 (2)	−0.0028 (13)	0.0011 (15)	−0.0025 (15)
C5	0.0397 (19)	0.0227 (16)	0.068 (3)	−0.0011 (15)	−0.0021 (18)	0.0024 (16)
C6	0.0390 (19)	0.0301 (18)	0.053 (2)	−0.0029 (15)	−0.0025 (17)	0.0164 (16)
C7	0.0245 (15)	0.0318 (17)	0.0393 (19)	−0.0044 (13)	−0.0043 (13)	0.0089 (14)
C8	0.0374 (18)	0.047 (2)	0.0331 (19)	−0.0060 (16)	−0.0018 (15)	0.0127 (16)
C9	0.0413 (19)	0.050 (2)	0.0238 (17)	−0.0036 (16)	−0.0022 (14)	−0.0013 (15)
C10	0.0293 (16)	0.0340 (17)	0.0303 (17)	−0.0014 (14)	−0.0010 (13)	−0.0011 (13)
C11	0.0210 (14)	0.0272 (15)	0.0314 (17)	−0.0034 (12)	−0.0024 (12)	0.0022 (12)
C12	0.0223 (14)	0.0261 (15)	0.0360 (18)	−0.0014 (12)	0.0002 (12)	−0.0006 (13)
C13	0.046 (2)	0.064 (3)	0.0282 (19)	−0.0095 (19)	−0.0032 (16)	−0.0023 (17)
C14	0.052 (2)	0.0385 (19)	0.037 (2)	0.0000 (17)	0.0006 (17)	−0.0080 (16)
O1	0.0481 (16)	0.097 (3)	0.0412 (17)	−0.0195 (17)	0.0000 (13)	0.0045 (16)
O2	0.063 (2)	0.083 (2)	0.059 (2)	−0.0335 (18)	0.0073 (16)	−0.0249 (18)
O3	0.0481 (17)	0.0449 (17)	0.101 (3)	0.0117 (14)	−0.0200 (17)	−0.0033 (17)
O4	0.0404 (17)	0.059 (2)	0.183 (5)	−0.0152 (16)	−0.040 (2)	0.027 (2)
C15	0.0266 (15)	0.0300 (16)	0.042 (2)	0.0048 (13)	−0.0038 (14)	0.0032 (14)
C16	0.0349 (17)	0.0307 (17)	0.042 (2)	−0.0006 (14)	−0.0031 (15)	0.0068 (14)
C17	0.0327 (17)	0.0369 (18)	0.0327 (18)	0.0018 (15)	−0.0001 (14)	0.0021 (14)

### Geometric parameters (Å, °)

**Table:**

Co1—O1	2.180 (3)	C7—C8	1.400 (5)
Co1—O2	2.145 (3)	C8—C9	1.361 (5)
Co1—O3^i^	2.229 (3)	C8—H8A	0.9300
Co1—O4^i^	2.126 (4)	C9—C10	1.399 (5)
Co1—N1	2.122 (3)	C9—H9A	0.9300
Co1—N2	2.103 (3)	C10—C14	1.489 (5)
Co1—C15	2.512 (4)	C11—C12	1.440 (5)
Co1—C17^i^	2.519 (3)	C13—H13A	0.9600
N1—C1	1.338 (4)	C13—H13B	0.9600
N1—C12	1.350 (4)	C13—H13C	0.9600
N2—C10	1.328 (4)	C14—H14A	0.9600
N2—C11	1.364 (4)	C14—H14B	0.9600
C1—C2	1.410 (5)	C14—H14C	0.9600
C1—C13	1.485 (5)	O1—C15	1.233 (4)
C2—C3	1.352 (6)	O2—C15	1.246 (5)
C2—H2A	0.9300	O3—C17	1.240 (4)
C3—C4	1.405 (5)	O3—Co1^ii^	2.229 (3)
C3—H3A	0.9300	O4—C17	1.226 (5)
C4—C12	1.411 (4)	O4—Co1^ii^	2.126 (4)
C4—C5	1.428 (5)	C15—C16	1.511 (5)
C5—C6	1.350 (6)	C16—C17	1.509 (5)
C5—H5A	0.9300	C16—H16A	0.9700
C6—C7	1.436 (5)	C16—H16C	0.9700
C6—H6A	0.9300	C17—Co1^ii^	2.519 (3)
C7—C11	1.398 (4)		
			
N2—Co1—N1	79.24 (10)	C9—C8—H8A	120.3
N2—Co1—O4^i^	119.53 (16)	C7—C8—H8A	120.3
N1—Co1—O4^i^	140.97 (13)	C8—C9—C10	120.8 (3)
N2—Co1—O2	136.06 (13)	C8—C9—H9A	119.6
N1—Co1—O2	92.45 (12)	C10—C9—H9A	119.6
O4^i^—Co1—O2	93.90 (16)	N2—C10—C9	120.9 (3)
N2—Co1—O1	89.80 (11)	N2—C10—C14	118.4 (3)
N1—Co1—O1	123.50 (12)	C9—C10—C14	120.7 (3)
O4^i^—Co1—O1	92.30 (13)	N2—C11—C7	122.8 (3)
O2—Co1—O1	59.10 (12)	N2—C11—C12	117.3 (3)
N2—Co1—O3^i^	97.95 (12)	C7—C11—C12	119.9 (3)
N1—Co1—O3^i^	86.71 (11)	N1—C12—C4	123.0 (3)
O4^i^—Co1—O3^i^	58.47 (12)	N1—C12—C11	117.8 (3)
O2—Co1—O3^i^	124.82 (14)	C4—C12—C11	119.2 (3)
O1—Co1—O3^i^	149.76 (13)	C1—C13—H13A	109.5
C1—N1—C12	119.0 (3)	C1—C13—H13B	109.5
C1—N1—Co1	128.3 (2)	H13A—C13—H13B	109.5
C12—N1—Co1	112.6 (2)	C1—C13—H13C	109.5
C10—N2—C11	118.9 (3)	H13A—C13—H13C	109.5
C10—N2—Co1	128.1 (2)	H13B—C13—H13C	109.5
C11—N2—Co1	113.0 (2)	C10—C14—H14A	109.5
N1—C1—C2	120.9 (3)	C10—C14—H14B	109.5
N1—C1—C13	118.2 (3)	H14A—C14—H14B	109.5
C2—C1—C13	120.9 (3)	C10—C14—H14C	109.5
C3—C2—C1	120.4 (3)	H14A—C14—H14C	109.5
C3—C2—H2A	119.8	H14B—C14—H14C	109.5
C1—C2—H2A	119.8	C15—O1—Co1	90.4 (2)
C2—C3—C4	120.0 (3)	C15—O2—Co1	91.7 (2)
C2—C3—H3A	120.0	O1—C15—O2	118.8 (3)
C4—C3—H3A	120.0	O1—C15—C16	120.9 (3)
C3—C4—C12	116.7 (3)	O2—C15—C16	120.3 (3)
C3—C4—C5	123.9 (3)	O1—C15—Co1	60.2 (2)
C12—C4—C5	119.4 (3)	O2—C15—Co1	58.6 (2)
C6—C5—C4	121.2 (3)	C16—C15—Co1	178.2 (2)
C6—C5—H5A	119.4	C17—C16—C15	113.5 (3)
C4—C5—H5A	119.4	C17—C16—H16A	108.9
C5—C6—C7	120.8 (3)	C15—C16—H16A	108.9
C5—C6—H6A	119.6	C17—C16—H16C	108.9
C7—C6—H6A	119.6	C15—C16—H16C	108.9
C11—C7—C8	117.2 (3)	H16A—C16—H16C	107.7
C11—C7—C6	119.4 (3)	O4—C17—O3	119.4 (4)
C8—C7—C6	123.4 (3)	O4—C17—C16	120.1 (3)
C9—C8—C7	119.4 (3)	O3—C17—C16	120.5 (3)

Symmetry codes: (i) *x*−1, *y*, *z*; (ii) *x*+1, *y*, *z*.
